# Towards Functional Annotation of the Preimplantation Transcriptome: An RNAi Screen in Mammalian Embryos

**DOI:** 10.1038/srep37396

**Published:** 2016-11-21

**Authors:** Wei Cui, Xiangpeng Dai, Chelsea Marcho, Zhengbin Han, Kun Zhang, Kimberly D. Tremblay, Jesse Mager

**Affiliations:** 1 Department of Veterinary and Animal Sciences, University of Massachusetts, Amherst, MA 01003, USA; 2 Department of Pathology, Beth Israel Deaconess Medical Center, Harvard Medical School, Boston, MA 02215, USA; 3 Harbin Institute of Technology, School of Life Science and Technology, Harbin 150080, China; 4 Laboratory of Mammalian Molecular Embryology, Institute of Animal Genetics and Reproduction, College of Animal Sciences, Zhejiang University, 866 Yuhangtang Road, Hangzhou 310058, China

## Abstract

With readily available transcriptome-wide data, understanding the role of each expressed gene is an essential next step. Although RNAi technologies allow for genome-wide screens in cell culture, these approaches cannot replace strategies for discovery in the embryo. Here we present, for the first time, a knockdown screen in mouse preimplantation embryos. Early mammalian development encompasses dynamic cellular, molecular and epigenetic events that are largely conserved from mouse to man. We assayed 712 genes for requirements during preimplantation. We identified 59 genes required for successful development or outgrowth and implantation. We have characterized each phenotype and revealed cellular, molecular, and lineage specific defects following knockdown of transcript. Induced network analyses demonstrate this as a valid approach to identify networks of genes that play important roles during preimplantation. Our approach provides a robust and efficient strategy towards identification of novel phenotypes during mouse preimplantation and facilitates functional annotation of the mammalian transcriptome.

Preimplantation development refers to the period from fertilization to implantation. The fertilized oocyte progresses through a number of cleavage divisions and three major transcriptional and morphogenetic events that lead to the first cell fate decision and development into a blastocyst stage embryo capable of implantation. The first of these dynamic events is the maternal-to-zygotic transition (MZT), which includes degradation of maternal mRNAs and replacement with zygotic transcripts, a dramatic reprogramming of gene expression which is required for successful embryo development^1^,^2^. In the mouse, zygotic genome activation (ZGA) is detectable in 1-cell stage embryos but occurs mostly at the 2-cell stage. MZT and ZGA are essential for continued development and establishment of totipotency^3^,^4^. The second event is embryo compaction, which initiates at 8-cell stage in mouse embryos. During compaction, blastomere morphology becomes flattened and biochemical changes to cellular metabolism, ion transport and cell-cell contacts result in early embryonic cells first resembling somatic cells^5^. Compaction is essential for developmental progression and segregation of the initial embryonic lineages^6^. Following compaction, the third critical event is blastomere allocation and cell fate determination. The blastomeres located inside of the morula give rise to the inner cell mass (ICM) from which the embryo proper is derived (Fig. 1A), whereas the outer blastomeres differentiate exclusively into the trophectoderm (TE) from which extra-embryonic tissues are derived^7^,^8^. Well-defined gene expression patterns occur within these two distinct lineages. For example, in the mouse embryo, the transcription factor Oct4 (also known as Pou5f1) is enriched in ICM and functions to promote pluripotency and inhibit differentiation, while the transcription factor Cdx2 becomes highly expressed in TE and influences epithelial differentiation^9^,^10^,^11^. Appropriate regulation and mutually exclusive localization of Oct4 and Cdx2 is critical for successful ICM/TE lineage separation and formation of a competent blastocyst^12^,^13^,^14^,^15^.

With the advent of large-scale transcriptome profiling and sequencing efforts that reveal gene expression dynamics during distinct developmental stages, tissues and in different species, understanding the role of each expressed gene is the next frontier. Although current RNA interference (RNAi) technologies allow for genome-wide knockdown studies in tissue culture models, these approaches cannot replace strategies for discovery in the embryo. Several studies have described transcriptome dynamics in preimplantation embryos^16^,^17^,^18^,^19^, however a large-scale functional annotation has not been reported.

Towards this goal, we established a robust and reliable RNAi based system in mouse embryos to study preimplantation development. All embryos in this study were cultured in 5% (low) oxygen to decrease oxidative stress and more faithfully recapitulate development *in utero*^20^. Under our conditions, control embryos after microinjection and culture show precisely the same morphological and developmental progression as embryos *in utero* (Fig. 1A) and the rate of blastocyst formation is greater than 90%^21^,^22^.

After knockdown (KD) by microinjection, embryos were assayed in several ways to maximize the identification of gene function (Fig. 1A). Embryos were first assessed for morphological development to blastocysts in order to determine if KD caused developmental failure. If KD embryos developed into blastocysts, then blastocyst potential was functionally assessed in a 3-day outgrowth (OG) assay, which has been used as a model of implantation^23^,^24^. OG assays can be used to ascertain the potential of both TE growth and ICM/ES colony formation. Under our conditions, approximately 60% of control blastocysts hatch from the zona pellucida, and attach to the culture plate forming a distinct ICM colony (Fig. 1A, far right, yellow dashed line) surrounded by robustly proliferating trophoblast cells (Fig. 1A, far right, green dashed line).

## Results

Microinjection of long double-stranded RNA (dsRNA) designed against specific transcripts has been used as a robust and specific approach to achieve gene silencing during preimplantation stages^25^,^26^,^27^,^28^ as there is no interferon response or significant off-target effect^29^. Since mammalian zygotes are available in relatively small numbers and microinjection is labor intensive, we sought to establish efficient screening strategies to overcome these technical challenges.

**Figure 1:**
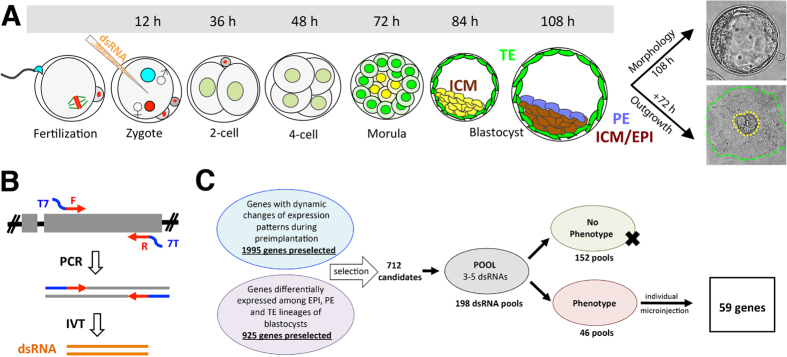
RNAi screen in preimplantation embryos. (**A**) Work flow and developmental progression of preimplantation embryos. (**B**) High efficiency bacterial cloning-free method to make dsRNA. (**C**) Candidates selection and pooling strategy to identify genes essential for preimplantation embryo development.

We first established an efficient dsRNA production protocol. dsRNAs were produced using an bacteria-free method (Fig. 1B). Briefly, PCR primers were designed to generate a 300–500 base pair amplicon – usually in the longest exon of each targeted gene allowing genomic DNA to be used as template for initial amplification. T7 promoter sequence was added to both forward and reverse primers. Following PCR amplification, *in vitro* transcription with T7 polymerase produced high quality, highly concentrated dsRNA suitable for microinjection (see methods for details).

**Figure 2:**
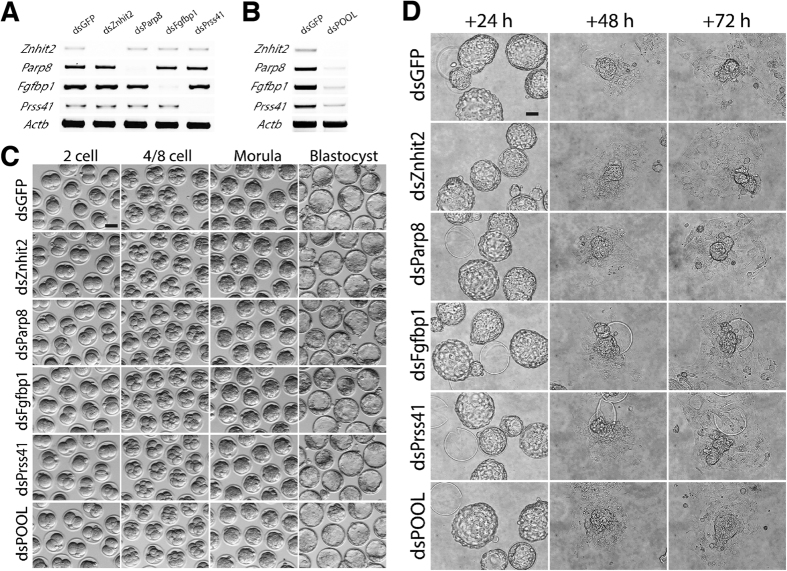
Efficient mRNA depletion after microinjection of dsRNA. (**A**) RT-PCR shows robust and specific transcript degradation after individual dsRNA injection. (**B**) RT-PCR results indicate simultaneous and efficient KD of 4 genes after pooled dsRNA injection. (**C**) No obvious developmental phenotypes after KD of these 4 genes – indicating a reliable screening system. (**D**) Similarly, knockdown of these genes does not inhibit blastocyst outgrowth when assayed. Scale bars, 50 μm.

We next determined a pooling strategy that allowed for reliable knockdown of multiple genes simultaneously (Figs 1C and 2). As shown in Fig. 1C, 3–5 different dsRNAs were injected together to knockdown multiple genes within the same embryo. Each pool of dsRNAs was injected into ~20 zygotes, and resulting embryos were assayed in multiple ways (developmental potential, morphology, and outgrowth). Pooled dsRNAs that were identified with phenotypes were then injected one at a time to determine the gene responsible, and to reproduce and validate each phenotype (Fig. 1C).

We used two strategies in order to compile a large list of candidate genes – both of which utilized available and published transcriptome studies. First, we took advantage of preimplantation microarray data (raw data)^17^,^18^,^19^ and extracted genes that were found to have at least a 5-fold change in mRNA expression during any two specific preimplantation stages. This analysis yielded 1995 candidates. We also mined available RNA-seq analyses for genes enriched in specific lineages – being highly expressed in epiblast (EPI), primitive endoderm (PE) or trophectoderm (TE)^16^. This approach added another 925 candidates. We then filtered these 2920 genes in multiple ways – largely to enhance the novelty of our candidates. Genes with known developmental functions and/or documented early lethal phenotypes during embryo development (Mouse Genome Informatics) were removed from the list. We also removed genes known or likely to be cell-lethal phenotypes base on documented functions (GeneCards Database www.genecards.org and PubMed Database), which accounted for a large number of our pre-selected candidates. These filtering steps left us with 748 candidate genes of which 712 were successfully screened by RNAi in preimplantation embryos (Fig. 1C, full lists in Supplementary Tables S1 and S2).

### Efficient KD after microinjection of pooled dsRNA

Figure 2 illustrates 4 different dsRNAs singly injected - each resulting in robust and specific knockdown of the target endogenous mRNA (examined by RT-PCR, Fig. 2A). As expected, KD of one gene does not disturb the expression of other transcripts, indicating the specificity of dsRNA mediated RNAi as previously reported. When these 4 dsRNAs are microinjected simultaneously, a similarly robust knockdown of each gene occurs (Fig. 2B). For all data presented, we use embryos injected with dsGFP as the negative control in order to stimulate the RNAi machinery and ensure identified phenotypes are specific for gene functions.

Despite mRNA knockdown of these 4 genes, embryos developed normally into blastocysts without obvious defects in morphology, developmental dynamics or blastocyst formation rate (Fig. 2C). Blastocysts were further assessed by three-day outgrowth (OG) assay. As shown in Fig. 2D, KD blastocysts hatched out of the zona pellucida by 24 hours, attached to the culture plate by 48 hours and formed ICM colonies with surrounding trophoblast cells at 72 hours. These results suggest these 4 transcripts are not functionally required during preimplantation and illustrate the efficacy of our approach. With confidence that our system would allow for identification of phenotypes specifically due to knockdown of individual genes, we injected dsRNAs designed against 712 genes in 198 experimental pools. As described below, we have identified 59 novel phenotypes during preimplantation development.

### Genes required for morula formation

Likely due to our removal of cell lethal genes from the candidate list, we identified only 4 phenotypes that resulted in cleavage stage embryo arrest. These phenotypes were observed after microinjection of dsRNA designed against *Dck*, *Itgae*, *Hist1h2a* and *Hist1h2b*. Each of these transcripts was found to be essential for morula formation. The majority of these KD embryos were arrested at 4–8 cell stage exhibiting morula failure after depletion of each transcript (Supplementary Figs S1 and S2), suggesting involvement of these genes in basic cellular events. *Dck* encodes deoxycytidine kinase which catalyzes phosphorylation of all four deoxynucleosides - essential for DNA replication. One recent study showed *DCK* plays a key role in cell proliferation^30^. *Hist1h2a* and *Hist1h2b* belong to histone cluster 1, the major histone gene locus also essential for DNA replication^31^. *Itgae* encodes the epithelial-cell-specific integrin alpha E, which mediates cell adhesion. Both *Hist1h2a* and *Hist1h2b* are members of gene families that are very well conserved. Therefore the dsRNAs target other family members as well suggesting that the morula failure phenotype is due to functional KD of many family members. As we are largely focused on lineage specification and blastocyst formation, we did not pursue these novel phenotypes further.

### Identification of 20 genes essential for blastocyst formation

**Figure 3:**
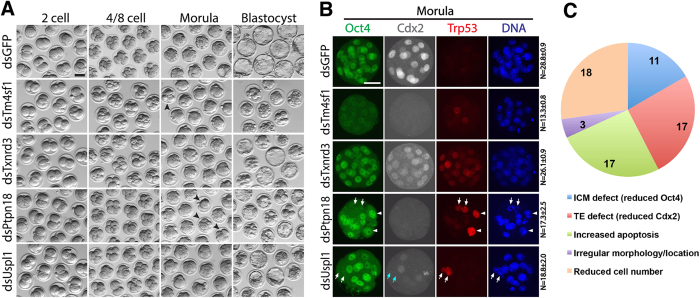
Twenty genes were identified essential for blastocyst formation. (**A**) KD Embryos were able to compact and develop into morulae while they failed to form blastocysts. Some embryos showed early phenotypes at morula stage (arrowheads). (**B**) KD morula embryos were characterized by IF. Arrows indicate the blastomeres lack of Oct4 and Cdx2 while with increased apoptosis. Arrowheads indicate the blastomeres arrested at earlier stage with bigger size nuclei and increased apoptosis. (**C**) 66 cellular/molecular defects were identified after characterizing these 20 genes involved in blastocyst failure. Scale bars, 50 μm.

We identified twenty genes that are required for successful blastocyst formation. These genes are *Actl6a*, *Gabpa*, *Hist1h3*, *Matr3*, *Mfng*, *Mxi1*, *Nop2*, *Pbrm1*, *Pnldc1*, *Ptpn18*, *Rpl7l1*, *Rrp7a*, *Rtn4*, *Sf3b1*, *Sf3b6*, *Supt6*, *Tm4sf1*, *Txnrd3*, *Uspl1*, and *Wdr74* (gene specific details provided in Supplementary Table S2). After depletion of each of these 20 transcripts, the majority of embryos were able to compact and develop into morphologically normal morulae with only a few arrested or delayed (Fig. 3A, arrowheads). However these KD morulae failed to progress into morphologically apparent blastocysts and remained as morulae or were visibly necrotic at 96 hours post-fertilization (when control embryos had formed expanded blastocysts, Fig. 3A; Supplementary Fig. S1). Four of these phenotypes are shown in Fig. 3 (others available in Supplementary Fig. S3). Since these KD phenotypes fail to form blastocysts, we examined morula stage embryos from each KD to investigate possible reasons for blastocyst failure. We performed immunofluorescence (IF) to examine localization and relative expression of Oct4 and Cdx2 critical determinants of ICM and TE, respectively. We also assayed for active Trp53 (p53) to reveal apoptotic blastomeres. Additionally we carefully counted the number of cells in all embryos to assess cleavage/development potential. The four gene KD shown in Fig. 3A,B are representatives of the range of results within this class of 20 phenotypes. Control dsGFP morula embryos showed robust and specific Oct4 and Cdx2 protein with most cells expressing either Oct4 or Cdx2 but not both at high levels (Fig. 3B, Oct4 shown as green, Cdx2 shown as white). Virtually no active p53 was detected in control embryos (Fig. 3B, red).

*Tm4sf1*-KD embryos (hereafter referred to as dsTm4sf1 embryos) exhibited globally reduced Oct4 and Cdx2, suggesting defects in both ICM and TE lineage specification, and a few apoptotic cells were observed in all dsTm4sf1 embryos. Although blastomeres of dsTm4sf1 morulae were able to compact without obvious abnormalities, blastomere number per embryo was significantly reduced compared to controls (Fig. 3B right column, dsGFP = 28.8 ± 0.9 cells/embryo, dsTm4sf1 = 13.3 ± 0.8 cells/embryo, *P* < 0.05). Combined, the absence of Oct4 and Cdx2 plus the reduction in cell number and only a few apoptotic cells suggest cell cycle arrest and possible block in global transcription or translation in the absence of Tm4sf1. Tm4sf1 is a member of transmembrane 4 superfamily of proteins – which have not been thoroughly characterized. One recent study in human breast cancer cells revealed that TM4SF1 stimulates cancer cell migration and invasion as well as inhibit apoptosis through PI3K/AKT/mTOR pathway^32^, offering the possibility that Tm4sf1 is required for blastomere growth and communication between blastomeres during preimplantation.

In contrast, dsTxnrd3 embryos maintained appropriate cell number with normal Oct4 positive blastomeres. However, no Cdx2 high blastomeres are observed in dsTxnrd3, with the majority of cells expressing low levels of Cdx2 (and high Oct4). Additionally, the majority of blastomeres in dsTxnrd3 embryos were p53 positive. *Txnrd3* has not been shown previously to have a role in early development. Our results are consistent with a previous study in intestinal epithelium revealing that the gene product thioredoxin reductase 3 is involved in defense against oxidative stress and has been implicated in cell proliferation and differentiation^33^. Together, these results suggest that in the absence of Txnrd3 function, blastomeres succumb to oxidative damage and undergo programmed cell death.

Whereas dsTxnrd3 embryos only showed globally increased apoptosis, dsPtpn18 and dsUspl1 embryos exhibited similar phenotypes with reduced total cell number, reduced number of Oct4 positive cells (Fig. 3B, arrows), an absence of Cdx2 high cells, and increased apoptosis (but only in a few cells). Intriguingly, both KD result in some blastomeres that were neither Oct4 nor Cdx2 positive – which does not normally occur. Notably, in dsPtpn18 embryos, blastomeres with nuclei of bigger size were apparent (Fig. 3B, arrowheads), suggesting defects during synthesis and/or mitosis. *Ptpn18* belongs to the protein tyrosine phosphatase (PTP) family that regulates multiple cellular processes including cell growth, differentiation, and mitosis. PTPN18 was recently found essential for HER2 (human epidermal growth factor receptor-2) activity^34^ which is broadly involved in both normal cell growth and tumorigenesis, suggesting Ptpn18 participates in a variety of cellular events required for embryo cleavage and blastomere growth.

Very little is known about *Uspl1*, which encodes ubiquitin specific peptidase like 1. Recent studies have demonstrated that human USPL1 is a cysteine protease belonging to ubiquitin-specific protease (USP) family that plays a key role in snRNA transcription, telomere integrity and cell proliferation^35^,^36^. A role in such fundamental cell viability requirements is consistent with an early developmental failure and suggests either that maternal protein allows dsUspl1 embryos to progress until morula stage or that other genes are redundant until blastocyst formation.

In this way we characterized all 20 blastocyst failure phenotypes with careful morphological assessment, cell counting and the presence of Oct4, Cdx2, and active p53 protein by IF (Supplementary Fig. S3, and Supplementary Table S3). For each phenotype we scored 5 characteristics based on the IF results: 1. ICM defect (reduced Oct4); 2. TE defect (reduced Cdx2); 3. Increased apoptosis; 4. Irregular morphology or cell location; 5. Reduced cell number per embryo. For example, dsTm4sf1 was scored with defects in ICM, TE, cell number and apoptosis, while dsTxnrd3 was scored only defective in p53+ cells. We analyzed all of the phenotypes together to ascertain if specific phenotypic features/defects were more prevalent than others. The most common defect observed was reduced total cell number (18/20 phenotypes), as well as increased apoptosis (17/20) and TE defect (reduced Cdx2 in 17/20). This summary analysis indicates that the genes we have identified with blastocyst failure phenotypes are likely involved in basic cellular events resulting in cell death and a defective TE lineage specification. Although this conclusion is not in itself surprising, identification of 20 new genes required for blastocyst formation greatly adds to our understanding of the molecular requirements for successful preimplantation development.

### Identification of 35 genes required for hatching and outgrowth

For all pooled dsRNA microinjections for which KD embryos developed into morphologically obvious blastocysts (with a visible blastocoel cavity), we subjected embryos to an additional 72-hour OG assay. OG assays have been used as a model for implantation, allowing for functional assessment of blastocysts^37^. Mouse blastocysts become expanded and hatch from the zona pellucida, attaining adhesion competence during the first 24 hours of OG culture. During the following 24 hours, hatched blastocysts attach to the culture dish and trophoblast cells begin to grow outward with surrounding primary trophoblast giant cells. Concurrently, as ICM cells proliferate, they pile up on one another creating a rudimentary ICM “stalk” like colony that is obvious by light microscopy. During the third 24 hours of OG culture, the ICM colony is taller and more obvious, surrounded by a monolayer of trophoblast that proliferates and expands in area^23^,^24^,^38^,^39^. Establishment of the ICM colony is essential for embryonic stem cell (ESC) derivation. Approximately 60% of control dsGFP blastocysts formed successful outgrowths with embryos hatching from the zona, attaching on the plate and establishing evident TE and ICM/ES lineages. This rate is similar to blastocysts isolated following in utero development. Pooled dsRNA injection blastocysts for which less than 30% of embryos performed as expected at each 24-hour period of OG assay were scored as positive for an OG phenotype.

Each individual dsRNA from pooled dsRNA that resulted in a phenotype was re-injected to determine which specific gene was responsible for the observed phenotype. In this way, 35 genes (Supplementary Table S2) were identified as indispensable for normal hatching and outgrowth. These genes include: *9130008F23Rik*, *Akap3*, *Ankrd7*, *Arhgdig*, *Asf1b*, *Bcor*, *Ccdc24*, *Ccdc62*, *Cmtm3*, *Coprs*, *Crxos*, *Ctr9*, *Fbll1*, *Hcfc1*, *Hs3st6*, *Lpar6*, *Ndufa2*, *Necab1*, *Pemt*, *Phf6*, *Plpp4*, *Ppp4r4*, *Slc25a34*, *Slc35e2*, *Smim14*, *St8sia6*, *Stmn3*, *Suds3*, *Suv39h1*, *Tbl1xr1*, *Tuba1*, *Ube2a*, *Zbed6*, *Zfp14*, *Zfp420*.

**Figure 4:**
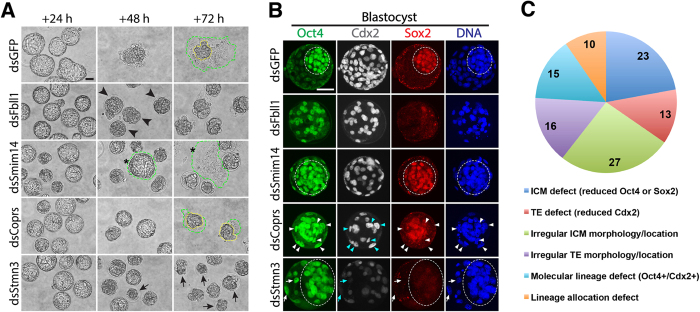
Thirty-five genes were identified essential for blastocyst outgrowth, 4 of 35 phenotypes are shown. (**A**) Blastocyst quality was functionally assessed by 3 days of OG. dsGFP control blastocysts hatch and attach to the plate after 24 hrs and 48 hrs culture, respectively; and finally form obvious ICM colony (yellow line) with proliferating trophoblast cells (green line) by 72 hrs. Examples shown are dsFbll1, dsSmim14, dsCoprs and dsStmn3 embryos. dsFbll1 blastocysts failed to hatch and were trapped inside of the zona (arrowheads). Most dsSmim14 embryos did not hatch and those that did hatch failed to form ICM colony (asterisk). dsCoprs embryos had delayed hatching and formed ICM colonies (yellow line) but with much smaller trophoblast OG areas (green line). dsStmn3 embryos degraded severely during the OG culture failing to hatch or proliferate (arrows). (**B**) KD blastocysts were characterized by IF. ICM cells (circled) in dsGFP control blastocysts are tightly arranged with robust expression of Oct4 and Sox2, and TE cells are uniformly arranged with specific expression of Cdx2. Arrowheads in dsCoprs point to Cdx2 positive cells in the outer layer that inappropriately remain Oct4 positive. Arrows in dsStmn3 indicate those outer layer cells possessing Oct4 high/Cdx2 low signals, which is opposite to control. Oct4 (green), Cdx2 (white), Sox2 (red), DAPI (blue). (**C**) Summary of 104 cellular/molecular defects that were identified among the 35 genes OG phenotypes. Scale bars, 50 μm.

Presented in Fig. 4 are a few examples of these phenotypes (complete results shown in Supplementary Figs S1 and S4). For example, dsFbll1 blastocysts failed to hatch out of the zona pellucida after 24 hours, and were visibly disorganized and/or proliferating but were trapped inside of the zona at 48 and 72 hours. This suggests that Fbll1 function is required for trophoblast-meditated hatching. Similarly, many dsSmim14 embryos failed to hatch, and those that did hatch failed to form an obvious ICM colony (Fig. 4A, asterisk). dsCoprs embryos exhibited delayed hatching and only did so after 48 hrs (as opposed to 24 hrs). dsCoprs ICM colonies were observed (Fig. 4A, yellow line) but these outgrowths had visibly smaller trophoblast outgrowth with very few TE cells (Fig. 4A, green line), suggesting TE was more severely affected than ICM after depletion of *Coprs*. Yet another phenotype observed was severely degrading/dying embryos of dsStmn3 embryos during the outgrowth assay (Fig. 4A, arrows), suggesting *Stmn3* is essential for cell survival – but only after blastocyst formation.

To explore the possible reasons for outgrowth failure and examine cell lineage allocation and organization, we analyzed Oct4, Cdx2, and Sox2 protein localization in KD blastocysts. We scored each of the 35 OG phenotypes as normal or defective for 6 characteristics: 1. ICM defect (reduced Oct4 or Sox2); 2. TE defect (reduced Cdx2); 3. Irregular ICM morphology/location; 4. Irregular TE morphology/location; 5. Molecular lineage defect (Oct4 and Cdx2 double positive cells); and 6. Lineage allocation defect (abnormal ratio of Oct4 positive and Cdx2 positive cells).

As shown in Fig. 4B, dsFbll1 blastocysts exhibited an obvious Sox2 reduction, suggestive of ICM defects. Additionally, ICM cells (Oct4 positive) were loosely associated and not aggregated together as in controls (Fig. 4B, circled). Furthermore, Cdx2 positive TE cells were not located uniformly on the outside of blastocysts and many cells remained Oct4 and Cdx2 positive. Combined, the lack of Sox2, the irregular location of both Oct4 and Cdx2 suggest that dsFbll1 blastocysts have both ICM and TE lineages defects.

The ICM of dsSmim14 embryos appears normal and appropriately organized with a tight cluster of Oct4/Sox2 double positive cells. However, the majority of outer blastomeres in dsSmim14 blastocysts were also Oct4 positive with very few Cdx2 positive cells. Combined with a failure to hatch from the zona pellucida (Fig. 4A), these results indicate a failure to specify functional TE lineage.

In dsCoprs embryo, Oct4 protein was found in numerous outer layer blastomeres that were also Cdx2 positive (Fig. 4B, arrowheads), indicating a failure to down regulate ICM transcriptional program in the TE cells and impaired molecular lineage specification. Additionally, only a small number of blastomeres were allocated to TE (Cdx2 positive) and these were not located regularly or uniformly, further supporting the notion of defective TE specification in the absence of Coprs function.

In dsStmn3 embryos, both Sox2 and Cdx2 were present in very few blastomeres, suggesting defects in both ICM and TE. Additionally, Oct4 signal was present in outer layer cells (Fig. 4B, arrows), indicating irregular ICM location or defective TE specification. This resulted in a much higher ratio of blastomeres being scored as ICM lineage (Oct4 positive) with scant TE cells (Cdx2 positive) scattered irregularly on the outer edge of dsStmn3 blastocysts.

Based on the scored criteria for each OG phenotype (Supplementary Fig. S4, Supplementary Table S4), 104 cellular/molecular defects were identified (Fig. 4C). The most common observation was defective ICM morphology/location (27/35 phenotypes), followed by ICM defect (reduced Oct4 or Sox2 in 23/35), suggesting that most of the genes identified are indispensible for ICM development and/or function. This is consistent with well-established studies showing that specification of TE is intimately linked to proper ICM allocation and function^9^,^40^.

### Expression patterns of the 59 genes essential for preimplantation

**Figure 5:**
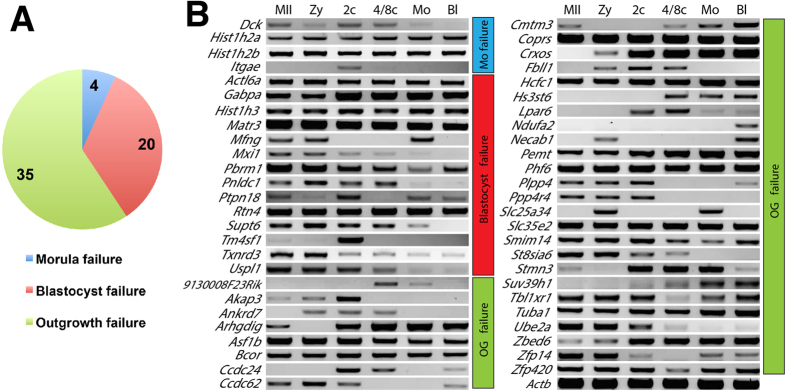
Total 59 genes identified as essential for preimplantation development. (**A**) Summary of these 59 phenotypes. (**B**) Expression patterns of each transcript in wild-type preimplantation embryos. MII, metaphase II oocyte; Zy, Zygote; 2c, 2-cell embryo; 4/8c, mix of 4- and 8-cell stage embryos; Mo, Morula; Bl, Blastocyst.

As presented above, 712 candidate genes were functionally assessed and 59 were identified essential for pre- or peri-implantation development. 4, 20 and 35 are functionally indispensable for proper formation of morula, blastocyst and outgrowth, respectively (Fig. 5A). In order to determine if the transcriptional profile of each gene might correlate with the observed phenotype, we characterized the normal expression pattern of each gene during preimplantation (Fig. 5B, and refs 21, 22, 27, 28, 41). Overall, we found no correlation of expression profile and phenotype. Although there are some genes with specific developmental windows of expression matching the timing of phenotype (*Itgae*, *Mfng*, *9130008F23Rik*, *Hs3st6*, *Ndufa2*), many are expressed at all stages examined, and there are several whose mRNA expression is incongruous with the observed phenotype. For example *Mxi1* and *Supt6* are not expressed in blastocysts – even though that is when they are functionally required. Similarly, *Akap3*, *Ankrd7*, *Fbll1*, *Ppp4r4*, *St8sia6* and *Ube2a* are not expressed in later stages but are required for successful OG. These discrepancies could be due to stable proteins with slow molecular turnover within cells^42^ or due to rapid transcriptional activation precisely when they are required^43^,^44^. Alternatively, these protein functions may be required for events temporally downstream in stages after their expression occurs as we have shown for other genes (*Suds3*^22^ and *Ctr9*^27^), which would suggest control of signaling cascades, transcriptional regulation or epigenetic functions.

### Gene ontology and network analysis

One major goal of the work presented was to screen a large set of genes that we did not select based on a molecular function of interest in order to identify unknown mRNAs and proteins involved in preimplantation development. We therefore sought to assess if there were particular GO terms associated with the 59 genes we identified. We also asked if we could infer novel pathways or gene networks that are required for successful preimplantation development based on the 59 genes that we did identify.

**Figure 6:**
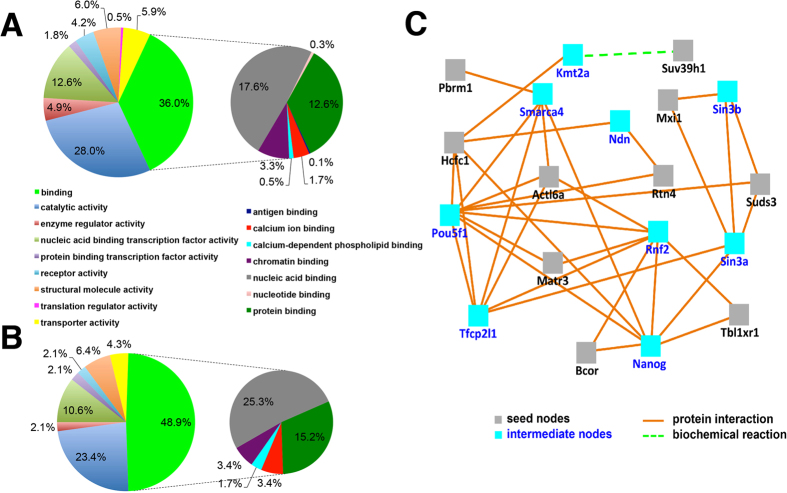
Gene Ontology (GO) and induced network modules analysis of screened genes. GO analysis was performed on 712 screen candidates (**A**) and 59 genes with phenotypes (**B**). Induced network modules analysis (**C**) was conducted with the 59 genes to assess relationships among them.

We first compared enrichment of molecular function GO terms between the full 712-gene list (Fig. 6A) and the 59 genes with preimplantation phenotypes (Fig. 6B). Only the smallest of the GO categories present in the initial list was absent in 59 phenotype genes (0.5% translation regulator activity) – likely due to the very small percentage representation. In other words, all categories of GO terms were still represented in the phenotypes at roughly similar representation. Among these terms/functions, the biggest change in percentage was “binding”, which increased from 36.0% in the initial list to 48.9% in the phenotype genes. Further analysis of sub-categories of binding revealed that similarly, all principal binding functions in large list are present in the phenotype list and that the increase in percentage of binding is largely due to an increase in “nucleic acid binding” (compare grey pie segments, right side of Fig. 6A and B), suggesting an enrichment for transcriptional regulators in genes with phenotypes. In summary, GO term analysis offered no striking outliers in functional categories within our screen results and suggests that preimplantation development equally requires all aspects of cellular and molecular function for success. Supporting this notion, previous studies have found essential roles during preimplantation for genes within each of these functional categories (including DNA/RNA/protein binding^45^,^46^, catalytic activity^47^,^48^, various transporter activity^49^,^50^,^51^).

Although the screen presented herein is not comprehensive, it provides clear pathway forwards towards assessment of all expressed genes during preimplantation which has not yet been accomplished. While our phenotype list of genes is small, we performed an “induced network module analysis”^52^ on the 59 genes we identified. As shown in Fig. 6C, this analysis revealed one major gene network stemming from 10 genes (seed nodes, grey squares) from our phenotype list (gene names in black, Fig. 6C). These nodes connect with each other by documented protein-protein interactions and also with 9 intermediate nodes that were not included in our screen list (blue gene names/squares, Fig. 6C). Importantly, each of these other genes (intermediate nodes) is also required for early embryonic development or neonatal survival^53^,^54^,^55^,^56^,^57^,^58^,^59^,^60^,^61^,^62^.

This analysis indicates the identification of a network of essential mammalian genes and suggests that even a limited screen can identify novel networks required during early development. Notably, due to the relatively un-annotated/unstudied bias of our gene lists, 49 of our genes with phenotypes were not found to have network amongst themselves using the current statistics in CPDB-mouse database^52^. With ever evolving annotation databases, our finding of so many novel phenotypes will contribute to these analyses in the future.

## Discussion

Since mammalian embryos lack interferon response^29^ and long dsRNAs are generally more efficient than single small interfering RNAs (siRNAs)^63^,^64^, dsRNA has been widely accepted as a robust and specific RNAi reagent to achieve gene silencing during early mammalian development^25^,^26^. dsRNAs are particularly useful when validated siRNAs are not available, since in our lab ~98% of dsRNAs result in KD greater than 80%. An additional benefit of dsRNAs is that they allow for rapid analysis of gene family members with similar sequence since one dsRNA can KD many closely related genes when designed appropriately. For example, in the screen presented herein, 4 of the dsRNAs intentionally result in KD of entire families of transcripts. *Hist1h2a*, *Hist1h2b*, *Hist1h3* and *Tuba1* were identified as essential for preimplantation development in our screen. When we designed RT-PCR assays that would intentionally amplify all of the family member transcripts (primers bind to conserved sequences), we observed consistent KD of all mRNAs simultaneously due to the single dsRNA injection (Supplementary Fig. S5).

To further confirm the specificity of dsRNA mediated RNAi, we microinjected commercial siRNAs against 6 genes with phenotypes from our screen (2 from each phenotypic category). Results showed that for each gene, two independent siRNAs that target different locations of same mRNA (Supplementary Table S5) resulted in identical developmental phenotype (Supplementary Fig. S6). Additionally, our previous experiments using dsRNA resistant mRNA^22^ and different siRNAs targeting separate locations of same mRNA^21^ also support the specificity of dsRNA mediated RNAi as a screening tool. Importantly, recent reports of gene knockout phenotypes are consistent with our screen induced phenotypes (*Actl6a*^65^, *Bcor*^66^, *Ctr9*^53^, *Gabpa*^67^, *Pbrm1*^68^, *Rtn4*^69^, *Supt6*^70^ and *Ube2a*^71^). Taken together these results all indicate that dsRNA mediated RNAi is a robust and specific approach for gene abrogation in early mammalian embryos.

Although the practical mechanics of microinjection and culture of mouse zygotes/preimplantation embryos are reproducible and robust, limited screens of early mammalian embryos have been performed. Generally, dsRNA is used to assess specific gene function after a gene of family has been identified for study. The major challenge of a large scale screen in embryos is the fact that mammalian zygotes are only available in relatively small numbers and microinjection is labor intensive. For these reasons, many groups defer to cell culture models where genome wide-screens are feasible due to the availability of robust tissue culture systems with automated processes. Here we have established a dsRNA pooling strategy suitable for preimplantation developmental studies (Fig. 1C), which allows for faithful KD of multiple genes within the same embryos. A similar method has recently been reported in mammalian oocytes^72^. While there are ongoing genome wide knockout efforts (https://www.komp.org), generation of null alleles will not adequately address functions during preimplantation due to retention of maternal RNA and protein that have accumulated during oocyte growth^73^. Although gene knockouts provide definitive functional assessment – the financial and temporal investment remains very large for each knockout allele.

The dsRNA approach described herein will complement ongoing knockout mouse consortium efforts and may offer important selection criteria for genes to be knocked out (preimplantation studies are not a priority of the KOMP). Rapid identification of genes required for preimplantation may also guide investigators to generate conditional knock-out alleles in order to study gene function in tissues/stages beyond preimplantation. Therefore we have made our complete screen results and gene lists available and searchable at http://blogs.umass.edu/jmager/.

Importantly, here we present results focused on obvious phenotypes that we identified during the screen (morula and blastocyst rate lower than 50% and OG rate lower than 30%). For blastocyst formation assessment, we adopted the visible blastocoel cavity as an unbiased standard but not the ratio of blastocoel cavity or the size of blastocyst. This may in part explain why more than half of the phenotype genes (35/59) are identified as OG failure genes (since small or shrunken blastocysts were still scored as “blastocysts”). Regardless, our analysis revealed defects in location and molecular identity of ICM and TE in each gene specific KD (Figs 3B and 4B, Supplementary Figs S3 and S4).

In summary, we present an efficient functional screening strategy in mammalian embryos and identify of 59 genes required for preimplantation development. Forty of these genes do not currently have published functional studies and nearly all have no documented role during early development. The wealth of novel results presented here highlights the importance of expanding this approach towards functional annotation of the mammalian genome.

## Methods

### Embryo recovery and culture

All animal experimental protocols were approved by the Institutional Animal Care and Use Committee of the University of Massachusetts, Amherst (approval No. 2013-009; 2016-0010). All procedures and methods were carried out in accordance with the approved guidelines and regulations. B6D2F1 female mice 8 to 10 weeks old were induced to superovulate with 5 IU pregnant mare serum gonadotropin (PMSG, Sigma-Aldrich, St. Louis, MO), followed 48 hr later by 5 IU human chorionic gonadotropin (hCG, Sigma-Aldrich, St. Louis, MO). Females were mated with B6D2F1 males and euthanized at 20 hr post-hCG injection for zygotes collection from the oviducts. Oviductal ampullae were dissected to release zygotes, and cumulus cells were removed by pipetting in M2 medium containing hyaluronidase (EMD Millipore, Billerica, MA). Zygotes were then washed in M2 medium (EMD Millipore, Billerica, MA) and cultured in KSOM medium (EMD Millipore, Billerica, MA) at 37 °C in a humidified atmosphere of 5% CO_2_/5% O_2_ balanced in N_2_. All cultured embryos were observed daily.

### Outgrowth assay

Blastocysts were collected and transferred gently into culture plate coated with 0.1% Gelatin (Sigma-Aldrich, St. Louis, MO) and cultured in DMEM (Lonza, Allendale, NJ) containing 10% fetal calf serum (Atlanta Biologicals, Flowery Branch, GA) and 1X GlutaMAX (Thermo Fisher, Agawam, MA). Outgrowth assay was conducted at 37 °C in a humidified atmosphere of 5% CO_2_ for 3 days and was observed daily.

### Batch primer design for T7 amplicons

FASTA files of cDNA sequences for genes of interest were acquired using Ensembl BioMart (GRCm38, Filters = Ensembl gene ID, Attributes = Sequences, cDNA sequences; Header information = Ensembl gene ID, Ensembl transcript ID). The longest exon for all genes of interest was identified using an in house perl script. Genes were then randomly assigned to pool groups. BatchPrimer3^74^ was used to design primers within the longest exon of each interest gene (minimum length = 250, optimum length = 350, longest length = 600). Genes with exons that did not meet primer requirements were designed individually using Primer3. T7 amplicons were BLASTed with Ensembl mouse cDNA database^75^ to make sure the overlap with other known transcripts is less than 20 bp. All T7 primer information is listed in Supplementary Table S1 and S2.

### Double-stranded RNA (dsRNA) preparation

DNA templates for T7-RNA polymerase mediated dsRNA production were amplified from genomic DNA or preimplantation embryo cDNA using primers containing T7 binding sequences followed by gene specific sequences for dsGFP (5′-TAATACGACTCACTATAGGGCACATGAAGCAGCACGACTT and 5′-TAATACGACTCACTATAGGGTGCTCAGGTAGTGGTTGTCG) or other dsRNAs (T7 primer information listed in Supplementary Table S1 and S2). PCR products were purified by QIAquick PCR Purification Kit (Qiagen, Hilden, Germany). *In vitro* transcription (IVT) was performed using a MEGAscript T7 Kit (Ambion, Waltham, MA) following the manufacturer’s instructions and TURBO RNase-free DNase was added to IVT product to degrade the DNA template. The *in vitro* transcribed sense and antisense single-stranded RNAs anneal during IVT (which is performed at 37 °C) to form dsRNA. dsRNA was then passed through NucAway Spin Columns (Ambion, Waltham, MA) to remove salt and unincorporated nucleotides. dsRNA was extracted with phenol/chloroform (Sigma-Aldrich, St. Louis, MO) and precipitated with 70% ethanol and resuspended in RNase-free water (Integrated DNA Technologies, Coralville, IA). The quality of dsRNA was confirmed by electrophoresis both after IVT and after final precipitation. The dsRNA concentration was measured using NanoDrop (Thermo Scientific, Waltham, MA) and dsRNA was diluted to around 3 μg/μl and stored at −80 °C until use.

### siRNA production and sequences

Both the scrambled control siRNA and gene specific siRNAs were purchased from Qiagen (Valencia, CA, USA). The sequences, target locations and catalog numbers of all siRNAs are listed in Supplementary Table S5. siRNAs were resuspended in RNase-free water to 100 μM solutions.

### Microinjection

Microinjection was performed in M2 medium using a Nikon inverted microscope equipped with a piezo-driven (Prime Tech, Japan) micromanipulator (TransferMan NK2, Eppendorf, Hamburg, Germany). A volume of 5–10 pl dsRNA (3 μg/μl) was microinjected into the cytoplasm of zygotes using a blunt-ended pipette of 6–7 μm in diameter. The same concentration and volume of dsGFP was injected as control in all experiments. For siRNA experiment, 5–10 pl (100 μM) of control or gene specific siRNA was microinjected into the cytoplasm of zygotes using same method as described above. After microinjection, zygotes were washed with M2 medium and cultured in KSOM medium at 37 °C in a humidified atmosphere of 5% CO_2_/5% O_2_ balanced in N_2_.

### RNA extraction and Reverse Transcription PCR (RT-PCR)

Total RNA extraction was performed with a Roche High Pure RNA Isolation Kit (#11828665001, Basel, Switzerland). cDNA was synthesized using iScript cDNA synthesis kit (Bio-Rad Laboratories, 170-8891, Hercules, CA). Specific primers were used for standard RT-PCR (*Actb*: 5′-GGCCCAGAGCAAGAGAGGTATCC and 5′-ACGCACGATTTCCCTCTCAGC; genes in Fig. 2: same as for the preparation of dsRNA T7 template; genes in final list: listed in Supplementary Table S2).

### Immunofluorescence (IF)

Embryos were fixed in 4% paraformaldehyde in PBS for 30 min, washed three times in washing buffer (PBS containing 0.1% Triton X-100), and permeabilized with PBS containing 0.5% Triton X-100 for 15 min. Embryos were then blocked for 1 hr in blocking buffer (PBS containing 10% fetal calf serum and 0.1% Triton X-100), and incubated overnight at 4 °C with primary antibodies diluted in blocking buffer. After three rinses with washing buffer, embryos were incubated for 1 hr with secondary antibodies (Alexa Fluor, Life Technologies, Carlsbad, CA) diluted 1:600 in blocking buffer. DAPI was used to stain nuclear DNA for morula blastomere counting. Embryos were washed three times with washing buffer and then mounted and observed with the Eclipse-Ti microscope (Nikon, Tokyo, Japan). Identical image capture settings were maintained for imaging same batch embryos. Primary antibodies used included: rabbit anti-Sox2, Abcam, ab97959, 1:200; goat anti-Oct4, Santa Cruz Biotechnology, sc-8628, 1:100; mouse anti-Cdx2, BioGenex, AM392-5M, 1:200; rabbit anti-Trp53, Cell Signaling Technology, #9284, 1:100.

### Gene Ontology analysis and induced network modules analysis

Molecular function of Gene Ontology analysis was performed using the PANTHER Classification System^76^. Induced network modules analysis was performed using CPDB-mouse database^52^ and Z value was set as 15.

### Phenotype scoring and reproducibility

After identification of dsRNA pools resulting in a phenotype, each single dsRNA microinjection experiment was repeated twice to validate the identified genes and developmental phenotypes. The number of injected embryos and percentage of embryos showing defects at different stages are listed in Supplementary Fig. S1. Through the RNAi screen, dsRNA injected embryos possessing morula/blastocyst rate lower than 50% and OG rate lower than 30% (approximately half the rate of the control group) were considered as phenotypes. Due to the nature of the screen, we pursued only the most robust phenotypes (those with at least 50% developmental failure – most with more than 80%), knowing that there may be subtle phenotypes that we did not analyze in detail. Therefore our complete screen results are available at http://blogs.umass.edu/jmager/.

## Additional Information

**How to cite this article**: Cui, W. *et al*. Towards Functional Annotation of the Preimplantation Transcriptome: An RNAi Screen in Mammalian Embryos. *Sci. Rep.* **6**, 37396; doi: 10.1038/srep37396 (2016).

**Publisher’s note:** Springer Nature remains neutral with regard to jurisdictional claims in published maps and institutional affiliations.

## Supplementary Material

Supplementary Table S1

Supplementary Table S2

Supplementary Table S3–S5 and Figure s1–S6

## References

[b1] Li L., Lu X. & Dean J. The maternal to zygotic transition in mammals. Molecular aspects of medicine 34, 919–938, doi: 10.1016/j.mam.2013.01.003 (2013). 23352575 PMC3669654

[b2] Latham K. E., Solter D. & Schultz R. M. Activation of a two-cell stage-specific gene following transfer of heterologous nuclei into enucleated mouse embryos. Molecular reproduction and development 30, 182–186 (1991). 1793594 10.1002/mrd.1080300303

[b3] Zhou L. Q. & Dean J. Reprogramming the genome to totipotency in mouse embryos. Trends in cell biology 25, 82–91, doi: 10.1016/j.tcb.2014.09.006 (2015). 25448353 PMC4312727

[b4] Schultz R. M. The molecular foundations of the maternal to zygotic transition in the preimplantation embryo. Hum Reprod Update 8, 323–331 (2002). 12206467 10.1093/humupd/8.4.323

[b5] Fleming T. P., Sheth B. & Fesenko I. Cell adhesion in the preimplantation mammalian embryo and its role in trophectoderm differentiation and blastocyst morphogenesis. Front Biosci 6, D1000–1007 (2001). 11487467 10.2741/fleming

[b6] Cockburn K. & Rossant J. Making the blastocyst: lessons from the mouse. The Journal of clinical investigation 120, 995–1003, doi: 10.1172/JCI41229 (2010). 20364097 PMC2846056

[b7] Zernicka-Goetz M., Morris S. A. & Bruce A. W. Making a firm decision: multifaceted regulation of cell fate in the early mouse embryo. Nature reviews. Genetics 10, 467–477, doi: 10.1038/nrg2564 (2009). 19536196

[b8] Arnold S. J. & Robertson E. J. Making a commitment: cell lineage allocation and axis patterning in the early mouse embryo. Nature reviews. Molecular cell biology 10, 91–103, doi: 10.1038/nrm2618 (2009). 19129791

[b9] Marikawa Y. & Alarcon V. B. Establishment of trophectoderm and inner cell mass lineages in the mouse embryo. Molecular reproduction and development 76, 1019–1032, doi: 10.1002/mrd.21057 (2009). 19479991 PMC2874917

[b10] Burton A. & Torres-Padilla M. E. Chromatin dynamics in the regulation of cell fate allocation during early embryogenesis. Nature reviews. Molecular cell biology 15, 723–734, doi: 10.1038/nrm3885 (2014). 25303116

[b11] Marcho C., Cui W. & Mager J. Epigenetic dynamics during preimplantation development. Reproduction 150, R109–120, doi: 10.1530/REP-15-0180 (2015). 26031750 PMC4529766

[b12] Wu G. . Initiation of trophectoderm lineage specification in mouse embryos is independent of Cdx2. Development 137, 4159–4169, doi: 10.1242/dev.056630 (2010). 21098565 PMC2990207

[b13] Niwa H. . Interaction between Oct3/4 and Cdx2 determines trophectoderm differentiation. Cell 123, 917–929, doi: 10.1016/j.cell.2005.08.040 (2005). 16325584

[b14] Strumpf D. . Cdx2 is required for correct cell fate specification and differentiation of trophectoderm in the mouse blastocyst. Development 132, 2093–2102, doi: 10.1242/dev.01801 (2005). 15788452

[b15] Paul S. & Knott J. G. Epigenetic control of cell fate in mouse blastocysts: the role of covalent histone modifications and chromatin remodeling. Molecular reproduction and development 81, 171–182, doi: 10.1002/mrd.22219 (2014). 23893501 PMC4276566

[b16] Yan L. . Single-cell RNA-Seq profiling of human preimplantation embryos and embryonic stem cells. Nature structural & molecular biology 20, 1131–1139, doi: 10.1038/nsmb.2660 (2013). 23934149

[b17] Zeng F., Baldwin D. A. & Schultz R. M. Transcript profiling during preimplantation mouse development. Developmental biology 272, 483–496 (2004). 15282163 10.1016/j.ydbio.2004.05.018

[b18] Hamatani T., Carter M. G., Sharov A. A. & Ko M. S. Dynamics of global gene expression changes during mouse preimplantation development. Developmental cell 6, 117–131 (2004). 14723852 10.1016/s1534-5807(03)00373-3

[b19] Wang Q. T. . A genome-wide study of gene activity reveals developmental signaling pathways in the preimplantation mouse embryo. Developmental cell 6, 133–144 (2004). 14723853 10.1016/s1534-5807(03)00404-0

[b20] Guerin P., El Mouatassim S. & Menezo Y. Oxidative stress and protection against reactive oxygen species in the pre-implantation embryo and its surroundings. Human reproduction update 7, 175–189 (2001). 11284661 10.1093/humupd/7.2.175

[b21] Cui W. . Nop2 is required for mammalian preimplantation development. Molecular reproduction and development 83, 124–131, doi: 10.1002/mrd.22600 (2016). 26632338 PMC4903073

[b22] Zhang K., Dai X., Wallingford M. C. & Mager J. Depletion of Suds3 reveals an essential role in early lineage specification. Developmental biology 373, 359–372, doi: 10.1016/j.ydbio.2012.10.026 (2013). 23123966

[b23] Qin J. . Regulation of embryo outgrowth by a morphogenic factor, epimorphin, in the mouse. Molecular reproduction and development 70, 455–463, doi: 10.1002/mrd.20225 (2005). 15685636

[b24] Sengoku K. . Requirement of nitric oxide for murine oocyte maturation, embryo development, and trophoblast outgrowth *in vitro*. Molecular reproduction and development 58, 262–268, doi: 10.1002/1098-2795(200103)58:3<262::AID-MRD3>3.0.CO;2-8(2001 ). 11170266

[b25] Svoboda P., Stein P., Hayashi H. & Schultz R. M. Selective reduction of dormant maternal mRNAs in mouse oocytes by RNA interference. Development 127, 4147–4156 (2000). 10976047 10.1242/dev.127.19.4147

[b26] Wianny F. & Zernicka-Goetz M. Specific interference with gene function by double-stranded RNA in early mouse development. Nature cell biology 2, 70–75, doi: 10.1038/35000016 (2000). 10655585

[b27] Zhang K., Haversat J. M. & Mager J. CTR9/PAF1c regulates molecular lineage identity, histone H3K36 trimethylation and genomic imprinting during preimplantation development. Developmental biology 383, 15–27, doi: 10.1016/j.ydbio.2013.09.005 (2013). 24036311 PMC4903072

[b28] Maserati M. . Wdr74 is required for blastocyst formation in the mouse. Plos one 6, e22516, doi: 10.1371/journal.pone.0022516 (2011). 21799883 PMC3143152

[b29] Stein P., Zeng F., Pan H. & Schultz R. M. Absence of non-specific effects of RNA interference triggered by long double-stranded RNA in mouse oocytes. Developmental biology 286, 464–471 (2005). 16154556 10.1016/j.ydbio.2005.08.015

[b30] Weng T. . Hypoxia-induced deoxycytidine kinase contributes to epithelial proliferation in pulmonary fibrosis. American journal of respiratory and critical care medicine 190, 1402–1412, doi: 10.1164/rccm.201404-0744OC (2014). 25358054 PMC4299646

[b31] Marzluff W. F., Gongidi P., Woods K. R., Jin J. & Maltais L. J. The human and mouse replication-dependent histone genes. Genomics 80, 487–498 (2002). 12408966

[b32] Sun Y., Xu Y., Xu J., Lu D. & Wang J. Role of TM4SF1 in regulating breast cancer cell migration and apoptosis through PI3K/AKT/mTOR pathway. International journal of clinical and experimental pathology 8, 9081–9088 (2015). 26464650 PMC4583882

[b33] Kipp A. P., Muller M. F., Goken E. M., Deubel S. & Brigelius-Flohe R. The selenoproteins GPx2, TrxR2 and TrxR3 are regulated by Wnt signalling in the intestinal epithelium. Biochimica et biophysica acta 1820, 1588–1596, doi: 10.1016/j.bbagen.2012.05.016 (2012). 22683372

[b34] Wang H. M. . The catalytic region and PEST domain of PTPN18 distinctly regulate the HER2 phosphorylation and ubiquitination barcodes. Cell research 24, 1067–1090, doi: 10.1038/cr.2014.99 (2014). 25081058 PMC4152746

[b35] Hutten S., Chachami G., Winter U., Melchior F. & Lamond A. I. A role for the Cajal-body-associated SUMO isopeptidase USPL1 in snRNA transcription mediated by RNA polymerase II. Journal of cell science 127, 1065–1078, doi: 10.1242/jcs.141788 (2014). 24413172 PMC3937775

[b36] Bermejo J. L. . Exploring the association between genetic variation in the SUMO isopeptidase gene USPL1 and breast cancer through integration of data from the population-based GENICA study and external genetic databases. International journal of cancer. Journal international du cancer 133, 362–372, doi: 10.1002/ijc.28040 (2013). 23338788

[b37] Armant D. R. Blastocysts don’t go it alone. Extrinsic signals fine-tune the intrinsic developmental program of trophoblast cells. Developmental biology 280, 260–280, doi: 10.1016/j.ydbio.2005.02.009 (2005). 15882572 PMC2715296

[b38] Qin J. . Effects of progranulin on blastocyst hatching and subsequent adhesion and outgrowth in the mouse. Biology of reproduction 73, 434–442, doi: 10.1095/biolreprod.105.040030 (2005). 15901638

[b39] Spindle A. I. & Pedersen R. A. Hatching, attachment, and outgrowth of mouse blastocysts *in vitro*: fixed nitrogen requirements. The Journal of experimental zoology 186, 305–318, doi: 10.1002/jez.1401860308 (1973). 4765353

[b40] Xenopoulos P., Kang M. & Hadjantonakis A. K. Cell lineage allocation within the inner cell mass of the mouse blastocyst. Results and problems in cell differentiation 55, 185–202, doi: 10.1007/978-3-642-30406-4_10 (2012). 22918807 PMC3469159

[b41] Maserati M., Dai X., Walentuk M. & Mager J. Identification of four genes required for mammalian blastocyst formation. Zygote 22, 331–339, doi: 10.1017/S0967199412000561 (2014). 23211737

[b42] Wu W., Hodges E., Redelius J. & Hoog C. A novel approach for evaluating the efficiency of siRNAs on protein levels in cultured cells. Nucleic acids research 32, e17, doi: 10.1093/nar/gnh010 (2004). 14739231 PMC373369

[b43] Wilson R. C. & Doudna J. A. Molecular mechanisms of RNA interference. Annual review of biophysics 42, 217–239, doi: 10.1146/annurev-biophys-083012-130404 (2013). PMC5895182 23654304

[b44] Agrawal N. . RNA interference: biology, mechanism, and applications. Microbiology and molecular biology reviews: MMBR 67, 657–685 (2003). 14665679 10.1128/MMBR.67.4.657-685.2003 PMC309050

[b45] Nestorov P., Hotz H. R., Liu Z. & Peters A. H. Dynamic expression of chromatin modifiers during developmental transitions in mouse preimplantation embryos. Scientific reports 5, 14347, doi: 10.1038/srep14347 (2015). 26403153 PMC4585904

[b46] Okamoto Y. . DNA methylation dynamics in mouse preimplantation embryos revealed by mass spectrometry. Scientific reports 6, 19134, doi: 10.1038/srep19134 (2016). 26750605 PMC4707515

[b47] Kim J. . Maternal Setdb1 Is Required for Meiotic Progression and Preimplantation Development in Mouse. Plos genetics 12, e1005970, doi: 10.1371/journal.pgen.1005970 (2016). 27070551 PMC4829257

[b48] Ma P. & Schultz R. M. HDAC1 and HDAC2 in mouse oocytes and preimplantation embryos: Specificity versus compensation. Cell death and differentiation 23, 1119–1127, doi: 10.1038/cdd.2016.31 (2016). 27082454 PMC4946893

[b49] Fong B., Watson P. H. & Watson A. J. Mouse preimplantation embryo responses to culture medium osmolarity include increased expression of CCM2 and p38 MAPK activation. BMC developmental biology 7, 2, doi: 10.1186/1471-213X-7-2 (2007). 17214902 PMC1781062

[b50] Cui W. . Control of spontaneous activation of rat oocytes by regulating plasma membrane Na+/Ca2+ exchanger activities. Biology of reproduction 88, 160, doi: 10.1095/biolreprod.113.108266 (2013). 23677981

[b51] Li Q. . Glucose metabolism in mouse cumulus cells prevents oocyte aging by maintaining both energy supply and the intracellular redox potential. Biology of reproduction 84, 1111–1118, doi: 10.1095/biolreprod.110.089557 (2011). 21270427

[b52] Kamburov A., Stelzl U., Lehrach H. & Herwig R. The ConsensusPathDB interaction database: 2013 update. Nucleic acids research 41, D793–D800, doi: 10.1093/nar/gks1055 (2013). 23143270 PMC3531102

[b53] Eppig J. T. . The Mouse Genome Database (MGD): facilitating mouse as a model for human biology and disease. Nucleic acids research 43, D726–736, doi: 10.1093/nar/gku967 (2015). 25348401 PMC4384027

[b54] Bultman S. . A Brg1 null mutation in the mouse reveals functional differences among mammalian SWI/SNF complexes. Molecular cell 6, 1287–1295 (2000). 11163203 10.1016/s1097-2765(00)00127-1

[b55] Voncken J. W. . Rnf2 (Ring1b) deficiency causes gastrulation arrest and cell cycle inhibition. Proceedings of the National Academy of Sciences of the United States of America 100, 2468–2473, doi: 10.1073/pnas.0434312100 (2003). 12589020 PMC151364

[b56] Martello G., Bertone P. & Smith A. Identification of the missing pluripotency mediator downstream of leukaemia inhibitory factor. The EMBO journal 32, 2561–2574, doi: 10.1038/emboj.2013.177 (2013). 23942233 PMC3791366

[b57] Mitsui K. . The homeoprotein Nanog is required for maintenance of pluripotency in mouse epiblast and ES cells. Cell 113, 631–642 (2003). 12787504 10.1016/s0092-8674(03)00393-3

[b58] Nichols J. . Formation of pluripotent stem cells in the mammalian embryo depends on the POU transcription factor Oct4. Cell 95, 379–391 (1998). 9814708 10.1016/s0092-8674(00)81769-9

[b59] Dannenberg J. H. . mSin3A corepressor regulates diverse transcriptional networks governing normal and neoplastic growth and survival. Genes & development 19, 1581–1595, doi: 10.1101/gad.1286905 (2005). 15998811 PMC1172064

[b60] David G. . Specific requirement of the chromatin modifier mSin3B in cell cycle exit and cellular differentiation. Proceedings of the National Academy of Sciences of the United States of America 105, 4168–4172, doi: 10.1073/pnas.0710285105 (2008). 18332431 PMC2393767

[b61] Yu B. D., Hess J. L., Horning S. E., Brown G. A. & Korsmeyer S. J. Altered Hox expression and segmental identity in Mll-mutant mice. Nature 378, 505–508, doi: 10.1038/378505a0 (1995). 7477409

[b62] Gerard M., Hernandez L., Wevrick R. & Stewart C. L. Disruption of the mouse necdin gene results in early post-natal lethality. Nature genetics 23, 199–202, doi: 10.1038/13828 (1999). 10508517

[b63] Barry G. . Gene silencing in tick cell lines using small interfering or long double-stranded RNA. Experimental & applied acarology 59, 319–338, doi: 10.1007/s10493-012-9598-x (2013). 22773071 PMC3557390

[b64] Wang J., Wu M., Wang B. & Han Z. Comparison of the RNA interference effects triggered by dsRNA and siRNA in Tribolium castaneum. Pest management science 69, 781–786, doi: 10.1002/ps.3432 (2013). 23526733

[b65] Krasteva V. . The BAF53a subunit of SWI/SNF-like BAF complexes is essential for hemopoietic stem cell function. Blood 120, 4720–4732, doi: 10.1182/blood-2012-04-427047 (2012). 23018638 PMC10627118

[b66] Cox B. J. . Phenotypic annotation of the mouse X chromosome. Genome research 20, 1154–1164, doi: 10.1101/gr.105106.110 (2010). 20548051 PMC2909578

[b67] Yu S. . GABP controls a critical transcription regulatory module that is essential for maintenance and differentiation of hematopoietic stem/progenitor cells. Blood 117, 2166–2178, doi: 10.1182/blood-2010-09-306563 (2011). 21139080 PMC3062326

[b68] Daxinger L. . An ENU mutagenesis screen identifies novel and known genes involved in epigenetic processes in the mouse. Genome biology 14, R96, doi: 10.1186/gb-2013-14-9-r96 (2013). 24025402 PMC4053835

[b69] Petrinovic M. M. . Neuronal Nogo-A negatively regulates dendritic morphology and synaptic transmission in the cerebellum. Proceedings of the National Academy of Sciences of the United States of America 110, 1083–1088, doi: 10.1073/pnas.1214255110 (2013). 23277570 PMC3549069

[b70] Dietrich J. E. . Venus trap in the mouse embryo reveals distinct molecular dynamics underlying specification of first embryonic lineages. EMBO reports 16, 1005–1021, doi: 10.15252/embr.201540162 (2015). 26142281 PMC4552493

[b71] Bruinsma C. F. . An essential role for UBE2A/HR6A in learning and memory and mGLUR-dependent long-term depression. Human molecular genetics 25, 1–8, doi: 10.1093/hmg/ddv436 (2016). 26476408

[b72] Pfender S. . Live imaging RNAi screen reveals genes essential for meiosis in mammalian oocytes. Nature 524, 239–242, doi: 10.1038/nature14568 (2015). 26147080 PMC4538867

[b73] Sanchez F. & Smitz J. Molecular control of oogenesis. Bba-Mol Basis Dis 1822, 1896–1912, doi: 10.1016/j.bbadis.2012.05.013 (2012). 22634430

[b74] You F. M. . BatchPrimer3: a high throughput web application for PCR and sequencing primer design. BMC bioinformatics 9, 253, doi: 10.1186/1471-2105-9-253 (2008). 18510760 PMC2438325

[b75] Flicek P. . Ensembl 2013. Nucleic acids research 41, D48–55, doi: 10.1093/nar/gks1236 (2013). 23203987 PMC3531136

[b76] Thomas P. D. . PANTHER: a library of protein families and subfamilies indexed by function. Genome research 13, 2129–2141, doi: 10.1101/gr.772403 (2003). 12952881 PMC403709

