# Supplementary material for: Towards Functional Annotation of the Preimplantation Transcriptome: An RNAi Screen in Mammalian Embryos
Source: Sci Rep. 2016 Nov 21;6:37396. doi: 10.1038/srep37396 (PMC5116644; doi:10.1038/srep37396)
Supplement: Supplementary Table S3–S5 and Figure s1–S6 [file srep37396-s3.pdf]

Supplementary Information:

Towards Functional Annotation of the Preimplantation Transcriptome: An RNAi Screen in Mammalian Embryos

Wei Cui[1], Xiangpeng Dai[2], Chelsea Marcho[1], Zhengbin Han[1,3], Kun Zhang[4], Kimberly D. Tremblay[1], Jesse Mager[1]*

[1] Department of Veterinary and Animal Sciences, University of Massachusetts, Amherst, MA 01003, USA
[2] Department of Pathology, Beth Israel Deaconess Medical Center, Harvard Medical School, Boston, MA 02215, USA
[3] Harbin Institute of Technology, School of Life Science and Technology, Harbin 150080, China
[4] Laboratory of Mammalian Molecular Embryology, Institute of Animal Genetics and Reproduction, College of Animal Sciences, Zhejiang University, 866 Yuhangtang Road, Hangzhou 310058, China

* Corresponding author: Jesse Mager, UMASS-Amherst, 661 North Pleasant Street, Amherst, MA 01003, USA; office: 413.545.7368; fax: 413.545.6326; Email: jmager@vasci.umass.edu

This part contains Supplementary Table S3-5 and Supplementary Figure S1-6.

Supplementary Table S3. Summary of defects after KD of genes essential for blastocyst formation

KD genes \ Phenotype	ICM defect (reduced Oct4) (11)	TE defect (reduced Cdx2) (17)	Increased apoptosis (17)	Irregular morphology /location (3)	Reduced cell number (18)	Note
Actl6a	+	+		+	+	
Gabpa			+	+	+	
Hist1h3	+	+	+		+	
Matr3		+			+	
Mfng		+			+	
Mxi1	+	+	+	+	+	
Nop2	+	+	+		+	Published[1]
Pbrm1	+	+	+		+	
Pnldc1	+	+	+		+	
Ptpn18	+	+	+		+	
Rpl7l1		+	+		+	Published[2]
Rrp7a		+	+		+	Published[2]
Rtn4	+	+	+		+	
Sf3b1		+	+		+	Published[2]
Sf3b6		+	+		+	Published[2]
Supt6		+	+		+	
Tm4sf1	+	+	+		+	
Txnrd3			+			
Uspl1	+	+	+		+	
Wdr74	+		+			Published[3]

Supplementary Table S4. Summary of defects after KD of genes essential for successful outgrowth

Phenotype / KD genes	ICM defect (reduced Oct4 or Sox2) (23)	TE defect (reduced Cdx2) (13)	Irregular ICM morphology/ location (27)	Irregular TE morphology/ location (16)	Molecular lineage defect (Oct4$^+$/Cdx2$^+$) (15)	Lineage allocation defect (10)	Note
9130008F23Rik			+		+	+	
Akap3	+	+	+	+	+		
Ankrd7	+		+		+		
Arhgdig	+		+		+		
Asf1b	+	+	+	+			
Bcor	+	+	+	+	+		
Ccdc24	+		+		+		
Ccdc62		+	+	+			
Cmtm3		+		+	+		
Coprs			+	+	+	+	
Crxos	+		+			+	
Ctr9	+				+		Published[4]
Fbll1	+		+	+			
Hcfc1	+	+	+	+			
Hs3st6	+	+					
Lpar6			+	+	+		
Ndufa2	+	+	+	+			
Necab1	+		+			+	
Pemt	+		+				
Phf6	+		+			+	
Plpp4			+		+		
Ppp4r4					+		
Slc25a34	+	+	+	+			
Slc35e2	+	+	+	+			
Smim14						+	
St8sia6			+				
Stmn3	+	+	+	+		+	
Suds3	+	+			+		Published[5]
Suv39h1	+	+	+	+			
Tbl1xr1	+		+			+	
Tuba1	+			+			
Ube2a						+	
Zbed6			+		+	+	
Zfp14			+		+		
Zfp420	+		+	+			

Supplementary Table S5. siRNA sequences, target locations and catalog numbers

Name	Sequence	Target location	Catalog number
Scrambled Control	5'-CAGGGTATCGACGATTACAAA	n/a	1027280
Dck siRNA1	5'-AAGCTTATATATAATTAACAA	2684:2704	SI00975163
Dck siRNA2	5'-AGCGGTGGAAATGTTCTTCAA	489:509	SI00975170
Itgae siRNA1	5'-TACCATGATGAGGAACTTCTA	692:712	SI00175343
Itgae siRNA2	5'-CACAAAGGAGGTGACCATGAA	2552:2572	SI02687293
Tm4sf1 siRNA1	5'-CCCATGGAGTATGGAACTACA	989:1009	SI04396532
Tm4sf1 siRNA2	5'-AACCTCGTTTACTGGCACTAA	455:475	SI04449235
Uspl1 siRNA1	5'-TTGAATAAAGTTAGAGATGAA	1274:1294	SI02768031
Uspl1 siRNA2	5'-AAGGAGGGATTTGCAACACTA	1633:1653	SI02770306
Fbll1 siRNA1	5'-CACGCAAGTCACCGCGTCAAA	272:292	SI04420416
Fbll1 siRNA2	5'-GCGCGTCACCGTGATGGAGAA	795:815	SI01305353
Stmn3 siRNA1	5'-CAGCACCGTATCTGCCTACAA	294:314	SI01436351
Stmn3 siRNA2	5'-TACCAGTATGGAGATATGGAA	391:411	SI01436365

Supplementary References

1 Cui, W. *et al.* Nop2 is required for mammalian preimplantation development. *Molecular reproduction and development* **83**, 124-131, doi:10.1002/mrd.22600 (2016).

2 Maserati, M., Dai, X., Walentuk, M. & Mager, J. Identification of four genes required for mammalian blastocyst formation. *Zygote* **22**, 331-339, doi:10.1017/S0967199412000561 (2014).

3 Maserati, M. *et al.* Wdr74 is required for blastocyst formation in the mouse. *PloS one* **6**, e22516, doi:10.1371/journal.pone.0022516 (2011).

4 Zhang, K., Haversat, J. M. & Mager, J. CTR9/PAF1c regulates molecular lineage identity, histone H3K36 trimethylation and genomic imprinting during preimplantation development. *Developmental biology* **383**, 15-27, doi:10.1016/j.ydbio.2013.09.005 (2013).

5 Zhang, K., Dai, X., Wallingford, M. C. & Mager, J. Depletion of Suds3 reveals an essential role in early lineage specification. *Developmental biology* **373**, 359-372, doi:10.1016/j.ydbio.2012.10.026 (2013).

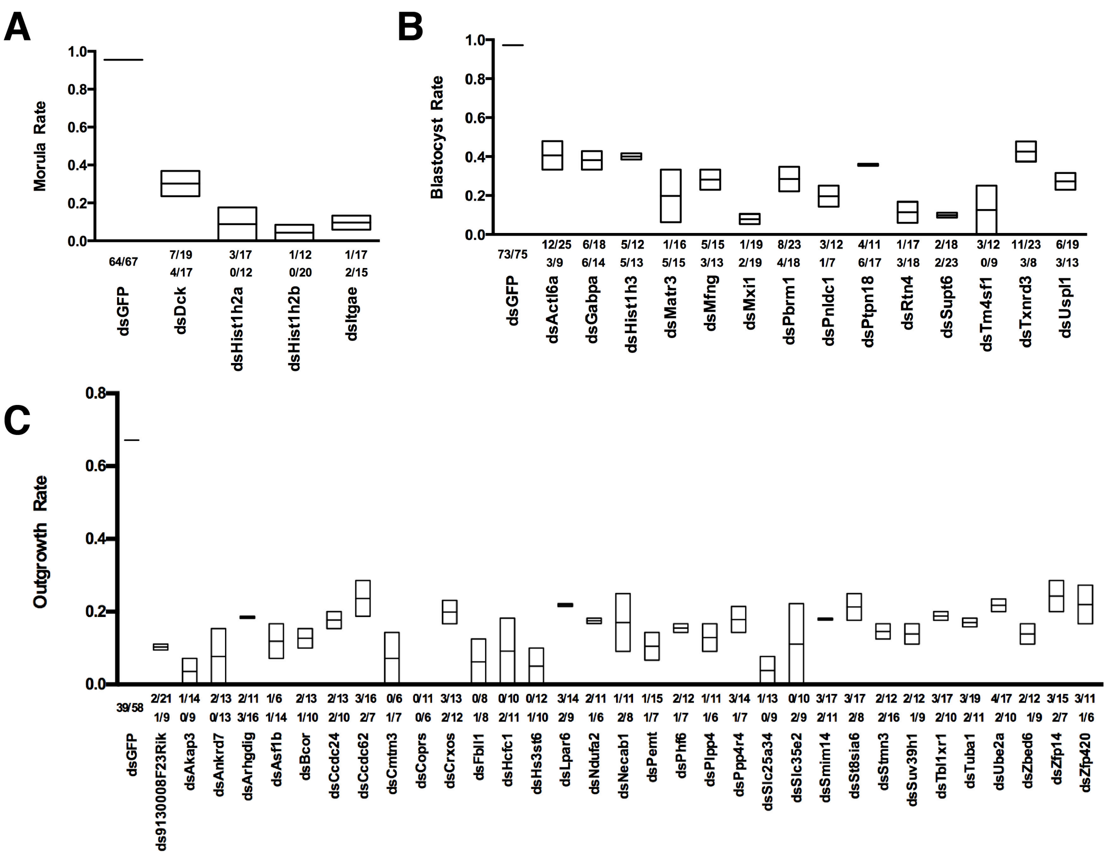

Supplementary Figure S1. Each single dsRNA microinjection was repeated twice to validate the identified genes and developmental phenotypes. (**A**) Number of injected embryos and percentage of embryos showing morula formation. dsGFP control data were from 4 randomly selected repeats. (B) Number of injected embryos and percentage of embryos showing blastocyst formation. dsGFP control data were from 4 randomly selected repeats. (C) Number of injected embryos and percentage of embryos possessing proper outgrowth. dsGFP control data were from 6 randomly selected repeats.

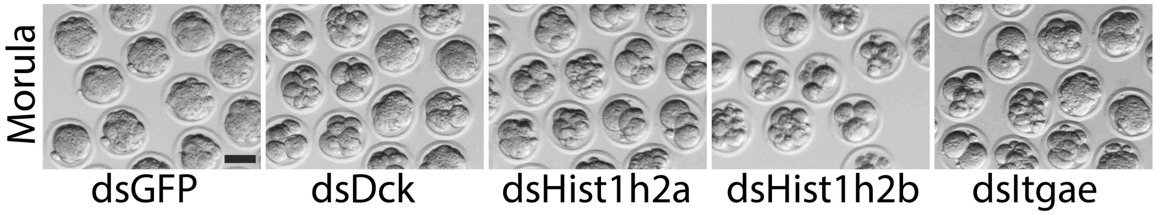

Supplementary Figure S2. Embryos were arrested at 4-8 cell stage showing morula failure after depletion of *Dck*, *Itgae*, *Hist1h2a* and *Hist1h2b*. Images taken 72 hours after fertilization and microinjection. Scale bar, 50 μm.

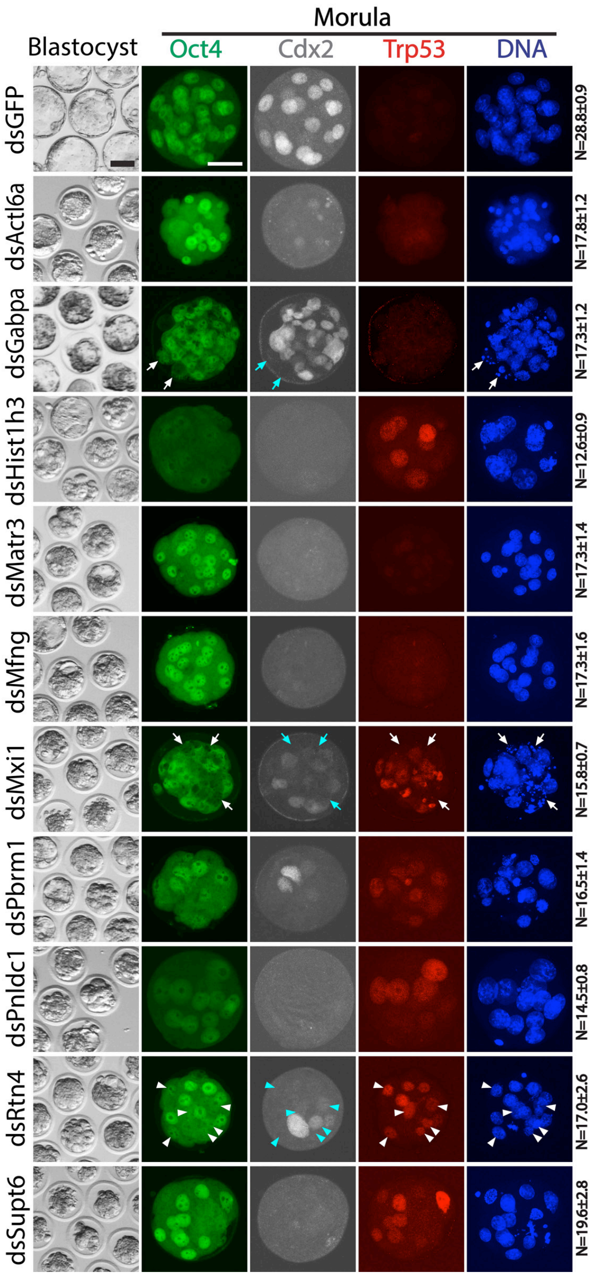

Supplementary Figure S3. Ten additional genes identified as essential for blastocyst formation. Left column shows the blastocyst formation failure after depletion of these transcripts by gene specific dsRNA injection. IF assays on KD-morula stage embryos show Oct4 (green), Cdx2 (white), p53 (red) and DNA (blue). Arrows in dsGabpa and dsMxi1 embryos indicate the blastomeres containing apoptotic bodies and without Oct4 or Cdx2 expression. Arrowheads in dsRtn4 embryo indicate the blastomeres lack of Oct4 and Cdx2 that are apoptotic. Detailed characterization listed in Supplementary Table 3. Scale bar, 50 μm.

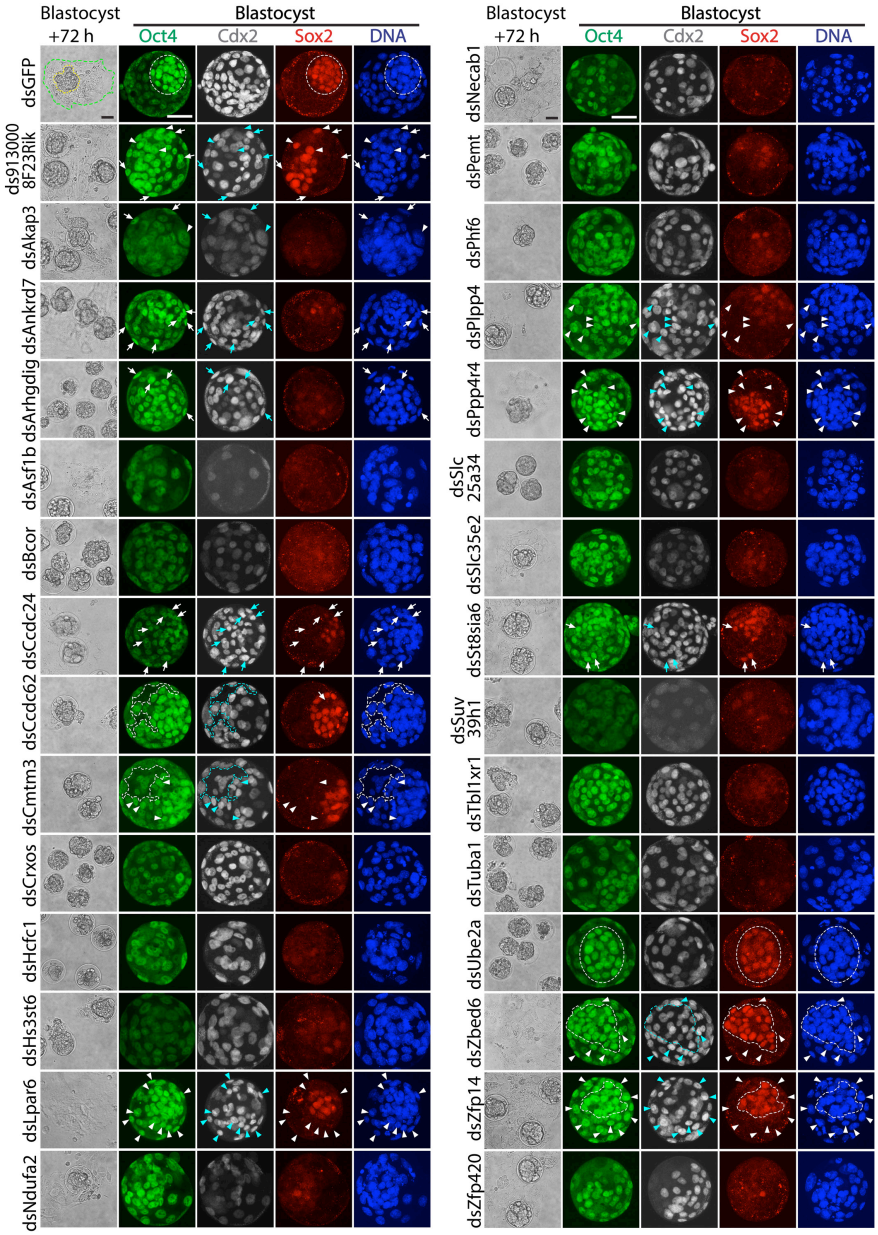

Supplementary Figure S4. Additional genes that were identified essential for OG. Left column shows the OG failure after depletion of these transcripts. Right columns show IF assays on the KD-blastocysts. Arrows in ds9130008F23Rik, dsAkap3, dsAnkrd7, dsArhgdig, dsCcdc24 embryos and arrowheads in dsCmtm3, dsLpar6, dsPlpp4, dsPpp4r4, dsZbed6, dsZfp14 embryos indicate blastomeres that are both Oct4 and Cdx2 positive. Arrowheads in ds9130008F23Rik embryo and arrow(s) in dsCcdc62 and dsSt8sia6 embryos indicate irregular ICM morphology/location. Arrowhead in dsAkap3 embryo indicates blastomere that is Oct4 and Cdx2 double positive and has a measurably larger nucleus suggesting delayed or arrested development. Irregular TE morphology/location areas in dsCcdc62 and dsCmtm3 embryos are marked by dashed lines. Dashed lines in dsUbe2a, dsZbed6, dsZfp14 embryos indicate the location of ICMs. Detailed characterization listed in Supplementary Tab. 4. Scale bar, 50 μm.

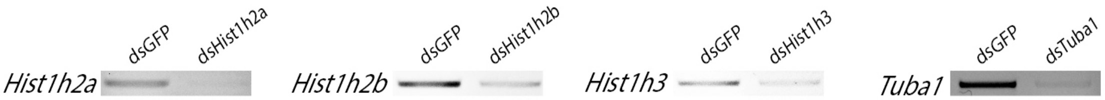

Supplementary Figure S5. RT-PCR results show efficient KD of multiple family members. Control embryos (dsGFP) and KD embryos were collected 48 hr post dsRNA injection. One embryo equivalent cDNA was used per RT-PCR reaction with primers that amplify all gene transcripts within each gene family. *Hist1h2a* primers amplify *Hist1h2ac,d,e,f,g,h,i,k,n,o, p. Hist1h2b* primers amplify *Hist1h2bc,e,f,h,j,l,n,q,r. Hist1h3* primers amplify *Hist1h3b,d,e,f,g,h,i. Tuba1* primers amplify *Tuba1a,b,c.*

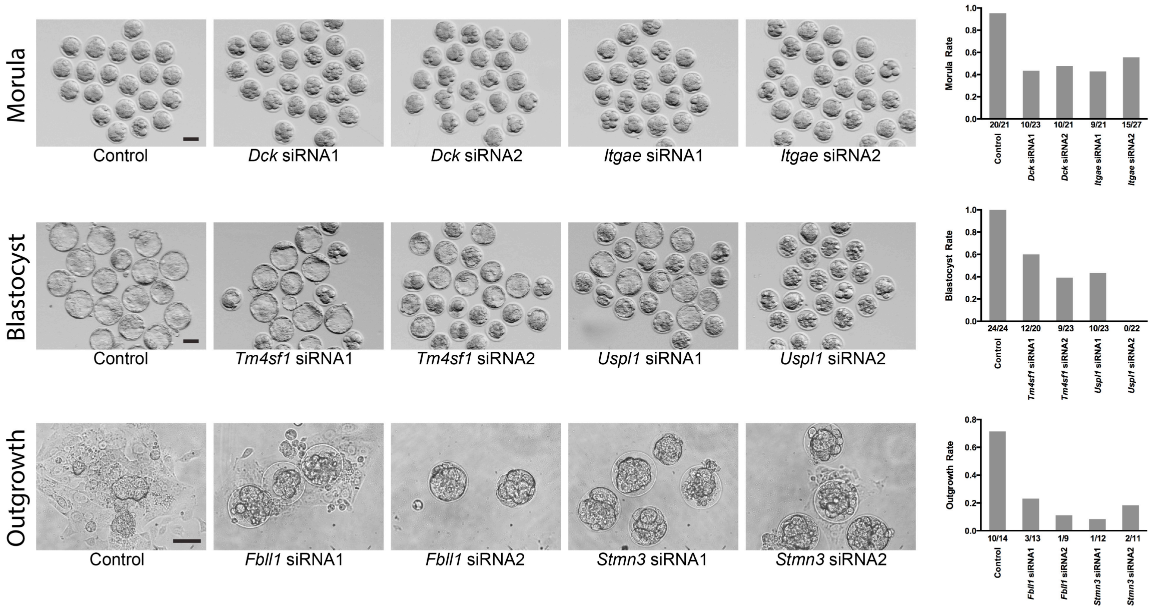

Supplementary Figure S6. Commercially purchased/designed independent siRNA sets of 6 randomly selected phenotype genes were microinjected into zygotes to confirm the specificity of dsRNA mediated RNAi as well as the identified genes and phenotypes. For each gene, two independent siRNAs that target different locations of same mRNA (listed in Supplementary Table S5 online) resulted in the same phenotype, which was also identical to the dsRNA induced phenotype. Number of injected embryos and percentage of embryos showing normal development were listed in the far right bar figures. Control embryos were injected with scrambled control siRNA. Scale bar, 50 μm.
